# Molecular insight in intrarenal inflammation affecting four main types of cells in nephrons in IgA nephropathy

**DOI:** 10.3389/fmed.2023.1128393

**Published:** 2023-03-09

**Authors:** Haidong Zhang, Zhenling Deng, Yue Wang

**Affiliations:** Department of Nephrology, Peking University Third Hospital, Beijing, China

**Keywords:** immunoglobulin a nephropathy, galactose deficient-immunoglobulin A1, complement, mesangial cell, endothelial cell, podocyte, tubular epithelial cell

## Abstract

Immunoglobulin A nephropathy (IgAN) is the most common primary glomerulonephritis and the leading cause of kidney failure in the world. The current widely accepted framework for its pathogenesis is the “multi-hit hypothesis.” In this review, we mainly discussed the intrarenal inflammation in IgAN, which is initiated by immune complex deposition with complement molecule activation, by focusing on four main types of cells in nephrons including mesangial cells, endothelial cells, podocytes, and tubular epithelial cells (TECs). Galactose-deficient IgA1 (Gd-IgA1)-containing immune complexes deposit in the mesangium and activate complement molecules and mesangial cells. Activation of mesangial cells by Gd-IgA1 deposition with enhanced cellular proliferation, extracellular matrix (ECM) expansion, and inflammatory response plays a central role in the pathogenesis of IgAN. Regional immune complex deposition and mesangial–endothelial crosstalk result in hyperpermeability of endothelium with loss of endothelial cells and infiltration barrier proteins, and recruitment of inflammatory cells. Podocyte damage is mainly derived from mesangial–podocyte crosstalk, in which tumor necrosis factor-α (TNF-α), transforming growth factor-β (TGF-β), renin-angiotensin-aldosterone system (RAAS), and micro-RNAs are the major players in podocyte apoptosis and disorganization of slit diaphragm (SD) related to proteinuria in patients with IgAN. In addition to filtrated proteins into tubulointerstitium and mesangial–tubular crosstalk involved in the injury of TECs, retinoic acid has been discovered innovatively participating in TEC injury.

## 1. Introduction

Immunoglobulin A nephropathy (IgAN) is the most common primary glomerular disease and the leading cause of kidney failure in the world with variable incidences in different countries (1). The difference in the estimated incidence of IgAN might be partly attributed to unsatisfactory systemic urine screening in some districts and disparities in current indications for kidney biopsy.

The most common clinical presentation of IgAN in adults is asymptomatic hematuria with varying degrees of proteinuria, with or without progressive kidney disease. Approximately 10–15% of adult patients with IgAN present as synpharyngitic macroscopic hematuria (2). Pathological lesion of IgAN is characterized by dominant or codominant Gd-IgA1 staining in the mesangium with ECM expansion and mesangial hypercellularity.

The “multi-hit hypothesis” is the current widely accepted framework for the pathogenesis of IgAN, in which a large amount of Gd-IgA1 characterized by the presence of galactose-deficient O-glycan in the hinge region is generated in the susceptible population after infection, and IgG autoantibodies targeting terminal N-acetylgalactosamine (GalNAc) residues following the deficiency of galactose in the hinge region on Gd-IgA1 are produced to form the IgG-Gd-IgA1 immune complex and deposit in glomeruli, mainly in the mesangium. These Gd-IgA1-containing immune complexes deposit in the mesangium, activate complement molecules, and initiate an intrarenal inflammatory response, which affects the four main types of cells in nephrons, namely, mesangial cells, endothelial cells, podocytes, and TECs.

Previous reviews have focused on clinical manifestation and therapeutic management in patients with IgAN (2), elucidated the genetic susceptibility of patients with IgAN (3), demonstrated the molecular structure and resources of Gd-IgA1 (4), and clarified the effect of mesangial–podocyte–tubular crosstalk in the pathogenesis of IgAN (5, 6). Trimarchi and Coppo (7) discussed podocyte damage in IgAN in detail. However, there was no review discussing the pathogenesis of IgAN by summarizing the alteration of four main types of cells in nephrons, and the contribution of intrarenal inflammation to renal injury in IgAN is still unclear. Therefore, we concluded the updated information on intrarenal inflammation in IgAN by focusing on the four main types of cells in nephrons, trying to draw a more comprehensive picture elucidating the pathogenesis of IgA nephropathy.

## 2. Gd-IgA1-containing immune complex deposition

Intrarenal inflammation is considered to be initiated by Gd-IgA1-containing immune complex deposition in the mesangium. Immunofluorescence in kidney samples of patients with IgAN reveals dominant or codominant mesangial Gd-IgA1 deposits (predominantly polymeric Gd-IgA1), accompanied by a variable degree of IgG and IgM. The majority of current evidence suggested that mesangial immune deposits are mainly derived from circulating Gd-IgA1-containing immune complexes. Deposited Gd-IgA1 is polyclonal, containing kappa and lambda light chains by immunofluorescence. Compared to the direct participation of Gd-IgA1 in the pathogenesis of IgAN, IgM might not participate in the pathogenesis of IgAN as it might be the result of entrapment of macromolecules secondary to glomerular injury. The structure of Gd-IgA1 and its corresponding synthesis process involved in IgAN was described by Knoppova et al. (4). High levels of Gd-IgA1 were generally considered to be produced by tonsillar lymphocytes (8) and plasma cells in the bone marrow and gut mucosa (3). Interestingly, plasma cells from gut mucosa could release dimeric IgA1, which could form polymeric IgA proteins, while IgA from the bone marrow is predominantly monomeric (9), suggesting Gd-IgA1 deposited in the mesangium with different forms might have different resources. However, the possibility that such polymeric IgA1 molecules are produced in the bone marrow of patients with IgAN has been proposed (10), and the location of polymeric IgA-producing cells in IgAN remains to be determined (11). Immunoglobulin might come from non-B cells is a new idea for the resources of immunoglobulin (12–14), and it has been reported that human mesangial cells could also produce IgA (15), which might be associated with the intrarenal inflammation in IgAN. We speculated that IgA produced and secreted by mesangial cells might at least contribute to Gd-IgA1 staining in kidney biopsies and the formation of circulating immune complexes in the plasma partly in patients with IgAN and might synergistically enhance the inflammatory response of mesangial cells together with Gd-IgA1 from B cells. Further experiments are urgently needed to confirm this speculation.

**Figure 1:**
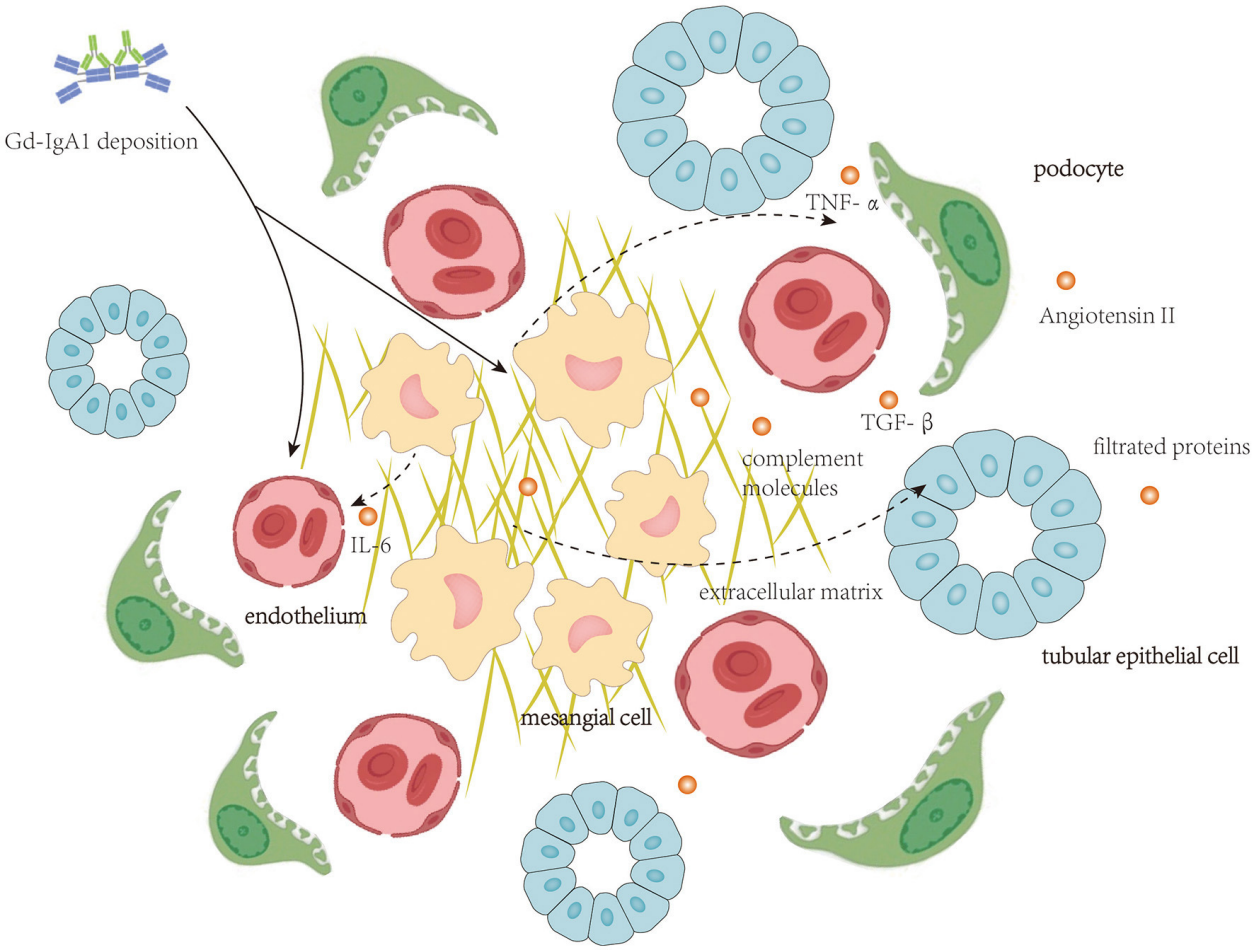
Local deposition of Gd-IgA1-containing immune complexes activate complement molecules and initiates intrarenal inflammation affecting mesangial cells, endothelial cells, podocytes, and tubular epithelial cells.

Autoantibodies targeting Gd-IgA1 in the blood of patients with IgAN are predominantly the IgG isotype (16), and IgG recognizes the hinge region of Gd-IgA1 with terminal GalNAc residues (4, 17). In patients with IgAN, IgG-bound Gd-IgA1 cannot be catalyzed by the liver (18), and therefore, these immune complexes remain in circulation for a prolonged period (19). Furthermore, the size of Gd-IgA1-IgG immune complex and polymeric Gd-IgA1 aggregated for their de-glycosylation is large, and these large molecules could not be cleared efficiently from the circulation and, thus, tend to deposit in the renal mesangium to initiate intrarenal inflammation. Clinical studies have revealed that circulatory levels of anti-Gd-IgA1 IgG antibodies correlate with disease severity (20). Intrarenal inflammation initiated by deposition of Gd-IgA1-containing immune complex affecting mesangial cells, endothelial cells, podocytes, and TECs is shown in Figure 1.

## 3. Activation of complement pathways

Complement molecules have been proven to participate in the pathogenesis of IgAN (21). Clinical studies assessing the serum and urinary levels of complement elements, and deposition in glomeruli have revealed the importance of these molecules in predicting renal outcomes (4).

Among the activation of the three complement pathways, the alternative pathway is considered an important player in the pathogenesis of IgAN as C3, properdin, and factor H were detected in the immune deposits in the kidney biopsies of patients with IgAN (21). *In vitro* experiments also indicated that IgA could activate alternative pathways (22). A single-nucleotide polymorphism (SNP) at position 1q32 in the factor H gene which leads to large deletion of complement factor H-related genes 1 and 3 (CFHR1 and CFHR3) was identified, positioned downstream of the factor H gene. Products of these two genes could bind C3 in a similar way as with factor H (23) to negatively regulate alternative pathways but with less efficiency compared to factor H. Absence of them would induce a stronger factor H-mediated inhibition of the alternative pathway, thereby loss of CFHR1 and/or CFHR3 from the specific SNP shows protective role in IgAN. Clinical study has shown that CFHR1 and CFHR3 deletion was associated with higher serum levels of factor H and C3, lower serum C3a levels, and less C3 mesangial deposition in patients with IgAN (24). Rare CFHR5 gene variants affecting FHR5 surface-binding regions to increase C3b binding capacity were also identified in patients with IgAN, resulting in greater complement-mediated injury and IgAN susceptibility (25).

Products of lectin pathway like mannose-binding lectin (MBL)-associated serine proteases (MASP) were detected in the glomerular deposits (2), suggesting the lectin pathway is also activated in IgAN. The interaction between MBL and IgA occurs between the carbohydrate recognition domain of MBL and specific glycosylation moieties on polymeric IgA (26). L-ficolin could also bind to Gd-IgA1 through GalNAc *via* exposed acetyl groups (27). Gd-IgA1-containing immune complex could stimulate mesangial cells to produce collectin 11, an initiator of the lectin pathway. Collectin 11 could deposit on the mesangial cell surface by interacting with IgA1, thus initiating the lectin pathway and accelerating C3 deposition on mesangial cells (28). Several clinical studies have confirmed the negative prognostic impact of the mesangial deposition of lectin pathway elements, including MBL, MASP-1 and MASP-2, L-ficolin, C4d, and C4-binding protein (16, 29). However, the importance of activation of the lectin pathway by binding to IgA in IgAN pathogenesis is still unknown.

Unlike the dominant roles of the alternative and lectin pathway in complement pathway activation in IgAN, C1q, the first step in the activation of the classical cascade, is usually missing in IgAN kidney biopsies, and C1q deposition was only found in only patients with 10% IgAN (30), suggesting classical pathway is less significant in IgAN compared to the other two pathways (4). However, the lack of C1q staining is not an absolute indication of the role of the classical pathway in IgAN since the half-life of C1q is very short and may disappear from the tissue quickly.

C3 in the common pathway is present in the mesangium in up to 90% of cases (2). The role of C3 in IgAN was well studied by several previous studies. It has been reported that Gd-IgA1 deposition could induce mesangial cells to express C3 under inflammatory conditions (IL-1 and TNF-α) in IgAN (31, 32), and C3 activation is greater with larger molecular weight aggregates (21), suggesting that C3 deposition in IgAN might be dependent on polymeric but not monomeric IgA. Yanagihara et al. (33) supported this speculation by figuring out that C3 activation requires IgG in Gd-IgA-containing immune complexes serving as a surface for C3 cleavage for the production of C3 breakdown products. Moreover, C3a was also considered to contribute to the production of ECM from mesangial cells by inducing them to a secretory phenotype (34). Furthermore, C3a and C5a could increase the production of chemokine (C-C motif) ligand 20 (CCL20) from mesangial cells and consequently augment Th9 cell recruitment and IL-9 levels, resulting in IgAN exacerbation (35). Terminal complement complex co-deposited with Gd-IgA1 in the mesangium was also detected in kidney biopsies (36). C5b9 induced production of interleukin-6 (IL-6) and TGF-β and mesangial cell apoptosis in a rat model of human mesangioproliferative glomerulonephritis (37, 38), while C5a receptor knockout mice have less proteinuria and reduced glomerular C3 and IgA deposition in another IgAN murine model (39).

## 4. Mesangial cell

Activation of mesangial cells by Gd-IgA1-containing immune complexes plays a central role in IgAN. Several kinds of immunoglobulin receptors are constitutively expressed on mesangial cells to recognize deposited Gd-IgA1-containing immune complex, including FcαR(CD89) (40), FcγR (41), transferrin receptors (TfR1/CD71) (42), β1,4-galactosyltransferase 1(β-1,4-GalT1) (43), and integrin α1/β1 and integrin α2/β1 (44). CD71 and β-1,4-GalT1 share common intracellular signaling pathways in mesangial cells (43). The relationship between CD71 expression on mesangial cells and the progression of IgAN was investigated by Jhee et al. (42). Mesangial cells could secrete soluble CD89 into the extracellular compartment, and these soluble CD89 could in turn upregulate the expression of CD71 after binding to Gd-IgA1 (45), which might strengthen the progression of IgAN. Cell culture experiments suggested circulating immune complex containing high levels of Gd-IgA1 with large molecular mass (>800 kDa) could activate mesangial cells by inducing cellular proliferation and overproduction of cytokines and components of ECM, whereas complex without Gd-IgA1 or Gd-IgA1 alone with low molecular mass (≤ 800 kDa) exhibits an inhibitory effect (46, 47). These large complexes bind to CD71 on mesangial cells' surface to activate mitogen-activated protein kinase/extracellular-signal-regulated kinase (MAPK/ERK) pathway and the phosphoinositide 3-kinase (PI3K)/protein kinase B (Akt)/mammalian target of rapamycin (mTOR) pathway (7, 47). Interestingly, the MAPK/ERK pathway is involved in pro-inflammatory cytokine secretion, and the P13K/Akt/mTOR pathway is involved in mesangial cell proliferation, respectively. In those IgA1-stimulated mesangial cells, SUMO1 protein, a protein that drives SUMOylation modification in post-translational modification, might contribute to mesangial cell proliferation by inhibiting their autophagy (48). Epigenetic factors might also participate in Gd-IgA1-induced mesangial cell activation. Dai et al. (49) demonstrated that histone deacetylase is upregulated in mesangial cells from patients with IgAN and subsequently activates TGF-β/recombinant mothers against decapentaplegic homolog 2/3 (Smad2/3) and Janus kinase 2 (Jak2)/signal transducer and activator of transcription 3 (Stat3) signaling pathways for cellular proliferation and ECM expansion. Gd-IgA1 could also induce mesangial cell ferroptosis by damaging mitochondria and increasing reactive oxygen species and malondialdehyde (50).

In addition to direct interaction between Gd-IgA and mesangial cells, there are several other factors enhancing mesangial cell proliferation, ECM production, and cytokine release from mesangial cells. B cell-activating factor belonging to the TNF family (BAFF), which is generally vital for B-cell survival, proliferation, and activation, could also target BAFF receptors on mesangial cells to promote proliferation through Akt activation (51) and enhance fibroblast factors expression like connective tissue growth factor (CTGF) and fibronectin (FN) in mesangial cells through tumor necrosis factor receptor-associated factor 6 (TRAF6)/NF-κB signaling pathway (52). ADAMTS5, one member of the metalloproteinase family, was upregulated in infiltrated monocytes in IgAN tubulointerstitium and glomeruli (but not in the tissue-resident macrophages), which affected multiple ECM proteins (53) including basement membrane components and basement membrane-binding integrins. Especially, ADAMTS5 could catalyze C3 in the mesangium into C3c and C3d and appeared to affect the interaction of IgA and associated proteins with cultured mesangial cells, thus limiting inflammation in the mesangium. Triggering receptor expressed on myeloid cells-1 (TREM-1), which was detected to be expressed on neutrophils and monocytes, is also expressed on mesangial cells to amplify the Gd-IgA1 deposition-induced inflammatory response through MARK/ERK and NF-κB signaling pathways (54). The mechanism of TREM-1-driven cell activation differs between neutrophils and monocytes (55), and the mechanism of TREM-1 exacerbating mesangial cell proliferation remains unknown. One of those hints suggested TREM-1 could also strengthen TLR-engaging inflammatory response by binding LPS (56), but it is still unknown whether TREM-2 is expressed by mesangial cells or not. RAAS was also activated in patients with IgAN. Angiotensin II subtype 1 receptor (AT1R) was downregulated in mesangial cells in response to enhanced intrarenal expression of angiotensin II (Ang II) (57). This regulation could be considered as a protective role of mesangial cells in controlling inflammation as Gd-IgA1-induced ERK1/2 activation is dependent on AT1R (47). GATA-binding protein 3 (GATA3), a transcriptional factor with essential roles in cell lineage commitment and differentiation, functions in mesangial cells maturation, and it is highly expressed by mesangial cells and renin-expressing cells of the juxtaglomerular apparatus in IgAN (58). Over-expression of GATA3 is associated with enhanced mesangial cell proliferation, sustained heavy proteinuria, and renal dysfunction in patients with IgAN.

In IgAN, mesangial cells could also participate in the renal immune response by acting as antigen-presenting cells (59), and mesangial cells in juxtaglomerular mesangial region play a role as a bridge connecting local immune response and systemic immune response by phenotypic alteration after Gd-IgA1 stimulation (60). Antigen–antibody immune complexes, complement components, and pathogen-associated molecular patterns (PAMPs) and/or damage-associated molecular patterns (DAMPs) could activate phagocytosis by mesangial cells *via* Fc receptors, C3 receptors, and Toll-like receptors (TLRs) expressed on the cell surface, respectively (61, 62). It has been reported that up to 15% of the mesangial cell population processes phagocytic function, and they are derived from bone marrow and belong to the family of mononuclear leukocytes (61). TLRs, which were traditionally considered to recognize PAMPs and/or DAMPs in innate immunity, were also reported to be involved in IgA-stimulated cytokines secretion from mesangial cells in IgA nephropathy. Activation of TLR4 on the mesangial cell surface can induce the release of a variety of chemokines and cytokines, including monocyte chemoattractant protein-1(MCP-1), IL-6, TGF-β, and TNF-α through myeloid differentiation factor 88 (MyD88)/NF-κB signaling pathway (62, 63). TLR3 on the mesangial cell surface is also involved in MCP-1-induced monocyte chemotaxis (64). Another key characteristic of mesangial cells as antigen-presenting cells is that they express a major histocompatibility complex (MHC) on the cell surface. Both MHC-II and MHC-I are expressed in mesangial cells (59). The expression of MHC-II on mesangial cells could be promoted by IFN-γ, and MHC-I could be promoted by TNF-α and IFN-γ (65, 66). Antigen presentation by mesangial cells to T lymphocytes is facilitated by intercellular cell adhesion molecule-1(ICAM-1) and CD80 on the mesangial cell surface. The activated mesangial cells drive the proliferation and differentiation of CD4+ T cells. CD4+ T cells activated by IFN-γ-stimulated mesangial cells undergo Th1 differentiation (67), and the IFN-γ from Th1 cells could feedback to mesangial cells to enhance expression of MHCI, MHCII, ICAM-1, inducible nitric oxide synthase (iNOS), chemokines, and Fc receptors, resulting in a stronger inflammatory response in the glomeruli (68). In addition, IL-1, IL-6, and TNF-α from mesangial cells in infection could induce Th22 cell differentiation and proliferation, and these TGF-β producing Th22 cells are recruited by CCL20, CCL22, and CCL27 secreted by mesangial cells to contribute to renal fibrosis (69). Th17 cells are also recruited by CCL20 and secreted by activated mesangial cells (70).

**Figure 2:**
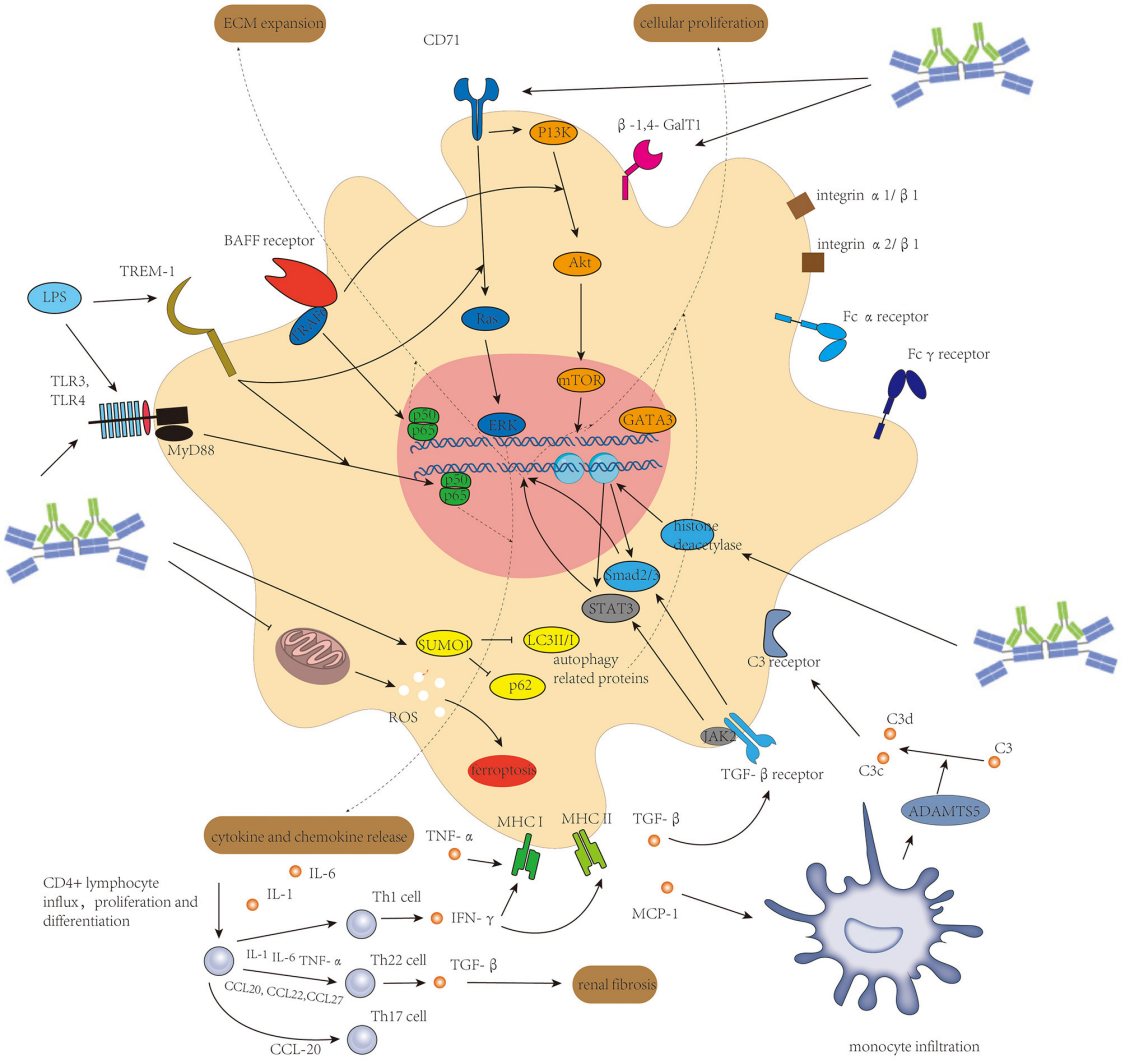
Activation of mesangial cells initiated mainly by Gd-IgA1-containing immune complexes and enhanced by several other factors induces cellular proliferation of mesangial cells, extracellular matrix expansion, and release of different kinds of cytokine and chemokine to connect intrarenal inflammation and systemic inflammatory response.

The associated molecular mechanism of mesangial cells in IgAN leading to cellular proliferation, ECM expansion, and acting as antigen-presenting cells is seen in Figure 2.

## 5. Endothelial cell

Clinical data have reported that damage of endothelium in IgAN with loss of endothelial cells is very common. Dissection of glomerular endothelial cells from the basal membrane, formation of endothelial cavitation, and insertion of the basal membrane was also observed in rat models with IgAN (71, 72).

Initially, damage to the endothelium was considered to be attributed to local Gd-IgA1 deposition. Gd-IgA1-containing immune complex deposition on endothelium could stimulate the expression of soluble vascular endothelial growth factor (sVEGF) receptor and soluble fms-like tyrosine kinase-1 (sFlt-1) by endothelial cells, leading to reduced proliferation ability and enhanced apoptosis of endothelial cells (73). Gd-IgA1-containing immune complexes mediate glycocalyx loss in endothelial cells (74). As glycocalyx covers the luminal surface of vascular endothelial cells, provides a barrier against free passage of proteins, and prevents the adherence of inflammatory cells to the endothelium (75), loss of glycocalyx results in hyperpermeability of the endothelium, deteriorating deposition of Gd-IgA1-containing immune complexes in the mesangium and might be partially attributed to proteinuria in patients with IgAN. Gd-IgA1-containing immune complexes could also increase the production of adhesion factors including vascular cell adhesion molecule-1 (VCAM-1), ICAM-1, and E-selectin to strengthen inflammatory cells recruitment and induce the production of pro-inflammatory cytokines such as TNF-α and IL-6 by glomerular endothelial cells for intrarenal inflammation exacerbation (74).

The effect of IL-6 (mainly from mesangial cells) in the formation of endothelial damage has been well studied by several groups. IL-6 increases the permeability of renal glomerular endothelial cells in IgAN by downregulating the expression of vascular endothelial cadherin *via* the trans-signaling pathway and inducing β-catenin phosphorylation on endothelial cells (76) through the classic pathway, respectively. In the trans-signaling pathway, IL-6 binds to soluble IL-6R to form the IL-6/IL-6R complex and thereafter binds to gp130 on endothelial cells to release MCP-1 by activating JAK/STAT3 and PI3K/AKT pathways simultaneously (77). IL-6 could also enhance endothelial cell proliferation and promote ICAM-1 expression on endothelial cells to strengthen monocyte–endothelial adhesion through downregulating miR-223 (78), in which NF-κB and STAT3 signaling pathways were activated. MiR-223 addition could inhibit NF-κB and STAT3 nuclear translocation, thereby alleviating endothelial cell proliferation, ICAM-1 expression, and monocyte adhesion on the endothelium. Another *in vitro* experiment demonstrated that elevated expression of endostatin, an endogenous angiogenesis inhibitor, and IL-6 with decreased cell proliferation activity was found in cultured endothelial cells after polymeric IgA stimulation (72). As direct stimulation of IL-6 enhances endothelial cell proliferation, it is reasonable to hypothesize that endostatin secreted by endothelial cells plays a self-protective role in cell proliferation while IL-6 from mesangial cells stimulated by Gd-IgA deposition enhances endothelial cell proliferation, and they collaboratively result in endothelial cell loss and hyperpermeability of endothelium finally.

**Figure 3:**
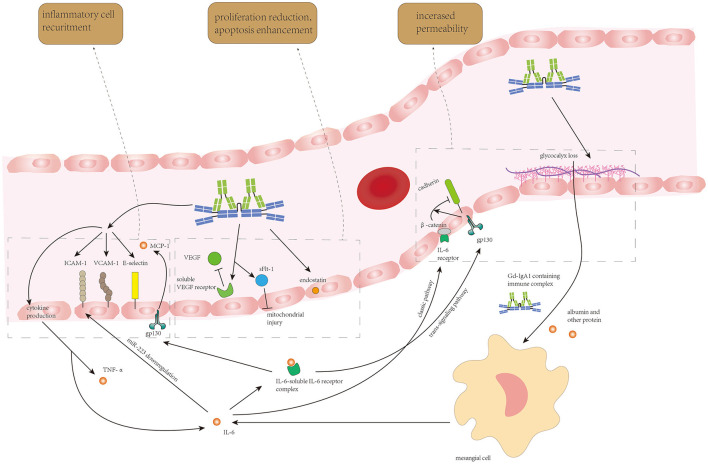
Circulating Gd-IgA1-containing immune complexes and IL-6 from endothelial autocrine and mesangial paracrine synergistically induce endothelial cell loss, increase in endothelium permeability, and recruitment of inflammatory cells to enhance Gd-IgA1 deposition and inflammatory response in the mesangium.

The endothelium damage resulting from Gd-IgA1-containing immune complex deposition and IL-6 from autocrine and paracrine is shown in Figure 3.

## 6. Podocyte

**Figure 4:**
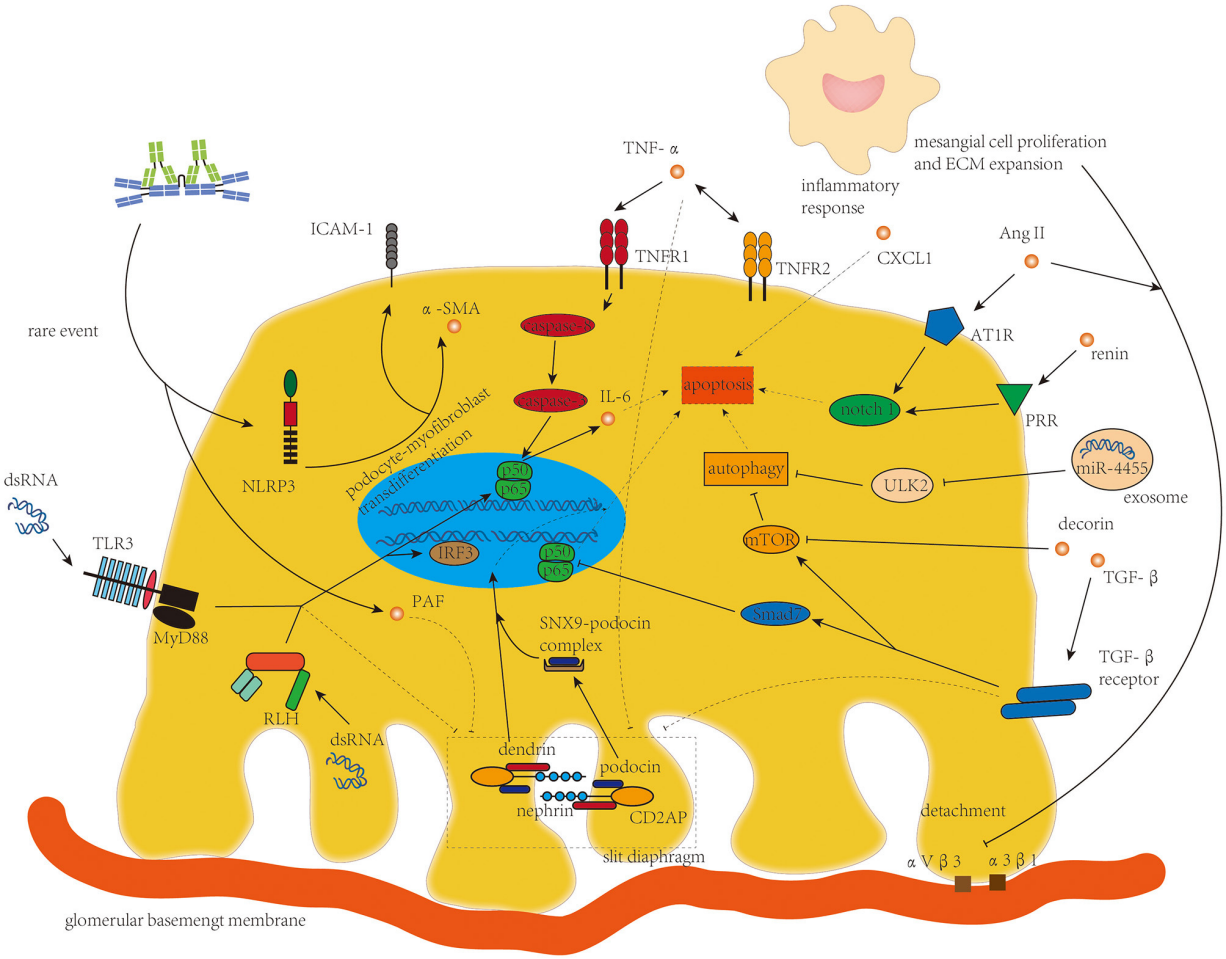
TNF-α, TGF-β, Ang II, and micro-RNAs from mesangial cells and dsRNA targeting toll-like receptor 3 and retinoic acid-inducible gene 1-like helicases are the main drivers of disorganization of the slit diaphragm and podocyte apoptosis in podocyte damage in IgAN.

Podocyte damage with increased glomerular permeability was found in part of patients with IgAN (79). Rather than the podocytopathy in lupus nephritis with diffuse foot process effacement and full nephrotic syndrome (80), podocyte damage in IgAN is typically localized and segmental and is correlated with proteinuria and decline in renal function in patients with IgAN (81). Process interfering with podocyte apoptosis and integrity of slit diaphragm (SD) is the main character of podocyte damage in IgAN (Figure 4). Podocyte detachment resulting from the lengthening of the mesangial axis (82) was also found in IgAN, in which the integrins αVβ3 and α3β1 on podocytes are involved (7).

Compared to mesangial cells, podocytes were rarely bound with Gd-IgA1 in IgAN because of the absence of known IgA receptors in podocytes (83). However, two *in vitro* experiments suggested that Gd-IgA1 deposition on the mouse podocyte cell line MPC-5 cells could induce podocyte apoptosis (84) and induce the expression of NOD-like receptor, pyrin domain-containing 3(NLRP3), an important sponsor after recognizing PAMPs and/or DAMPs in innate immunity, and initiate podocyte-macrophage transdifferentiation (PMT) to promote inflammation and renal fibrosis with an increased level of ICAM-1 and α-smooth muscle actin (α-SMA) (85). In these two *in vitro* experiments, aggregated IgA1 incubated from monomeric IgA1 manually was used to substitute polymeric IgA1 from patients with IgAN to stimulate MPC-5 cells. Different glycosylation moieties between aggregated IgA1 and polymeric IgA1 and different species of the cells might be the reason why Gd-IgA1 deposited on podocytes *in vitro* but not *in vivo*.

The damage of podocytes in IgAN has been supposed to originate mainly from altered mesangial–podocyte crosstalk (4), in which elevated amount of TNF-α and TGF-β is released from mesangial cells and reduces expression of nephrin, erzin, and podocin in podocytes (83). Increased synthesis of platelet-activating factor (PAF) in podocytes induced by IgA deposition acts as a secondary mediator in nephrin reduction (86). TNF-α from mesangial cells increases TNF-α synthesis and expression level of TNF receptor 2 (TNFR2) on podocytes, which enhances IL-6 synthesis to induce podocyte apoptosis (87) and activates the inflammatory response of podocytes (57) *via* TNFR1 and TNFR2, respectively. TNF-α-induced podocyte apoptosis was also detected to be associated with increased expression of caspase-3 in renal tissue of mouse model (88), suggesting TNF-α might induce podocyte apoptosis through TNFR1-/caspase-8/caspase-3 death pathway. Another cell culture experiment demonstrated that TNF-α might induce podocyte apoptosis and foot process effacement through the NF-κB signaling pathway (7). However, direct evidence confirming the mechanism of TNF-α in inducing apoptosis of podocytes is still needed. The dendrin was restored in the nucleus in injured podocytes to modulate apoptosis of podocytes after TGF-β stimulation and the podocyte apoptosis induced by TGF-β was significantly alleviated in dendrin knockdown podocytes (89), suggesting the role of translocational dendrin in enhancing podocyte damage (90). Podocin, another constructing protein of SD, is also endocytosed in IgAN, which is facilitated by the newly identified protein sorting nexin 9 (SNX9) (91). Smad7 plays as an amplifier in TGF-β-induced podocyte apoptosis by inhibiting nuclear translocation and transcriptional activity of NF-κB, while decorin, a small proteoglycan secreted by mesangial cells, antagonizes the effect of TGF-β1 through the mTOR pathway to inhibit podocyte apoptosis (92). However, decorin is sequestered in the accumulated mesangial matrix in IgAN and the level of podocyte-encountered decorin decreases, leading to enhanced podocyte apoptosis by TGF-β (93).

RAAS and micro-RNA are another two players in the mesangial–podocyte crosstalk in podocyte damage. Prorenin receptor (PRR) and angiotensin II type 1 receptor (AT1R) expressions on podocytes are enhanced by humoral mediators released by activated mesangial cells to aggravate podocyte apoptosis through the notch 1 signaling pathway (5, 94). Ang II from Gd-IgA-stimulated mesangial cells could modulate α3β1 integrin expression on podocytes to reduce their adhesiveness (95). Yu et al. (96) recently reported that MiR-4455 derived from mesangial cells is upregulated and transferred to podocytes, directly reducing expression of Unc-51-like autophagy activating kinase 2 (ULK2) and inducing podocyte injury through inhibiting autophagy.

DsRNA from virus replication contributes to glomerular injury and might be associated with the progression of IgAN (3). Podocytes express TLR3 and retinoic acid-inducible gene 1 (RIG-I)-like helicases (RLHs) to recognize the extracellular and cytosolic dsRNA, respectively. After binding to dsRNA, these two pathways induce phosphorylation of transcription factor interferon regulatory factor 3 (IRF3) and NF-κB and their translocation to the nucleus for protein synthesis. DsRNA also suppresses podocyte cell migration, which alters the expression of SD-constructing proteins including nephrin, podocin, and CD2-associated protein (CD2AP) to enhance proteinuria in patients with IgAN (97).

## 7. Tubular epithelial cell

**Figure 5:**
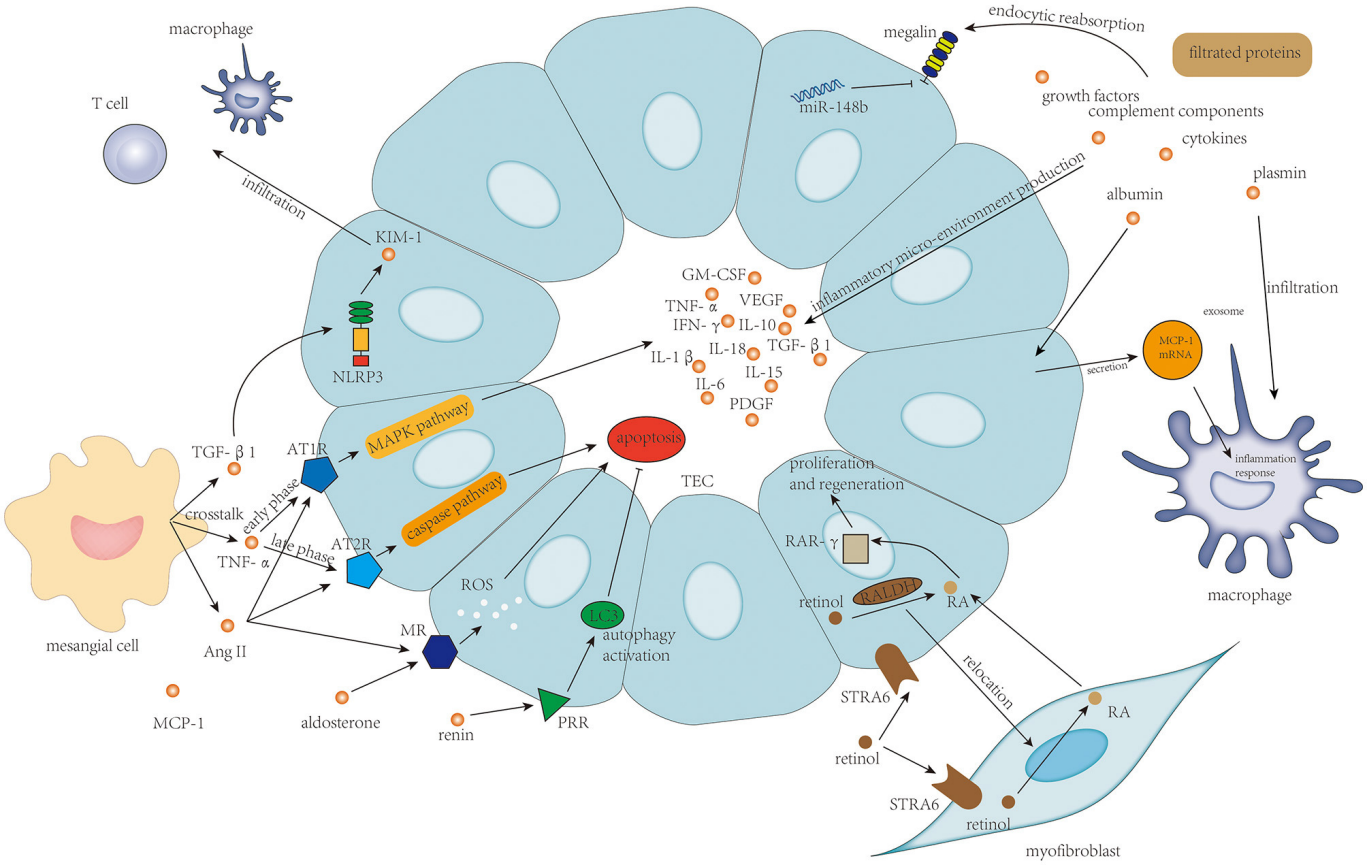
Protein filtration from endothelium damage and podocyte damage, and TNF-α, TGF-β, and activation of the renin–angiotensin–aldosterone system in mesangial–tubular crosstalk and the newly discovered retinol metabolism are the main determinants of survival of tubular epithelial cells and tubulointerstitial inflammatory response.

Limited studies were focused on TEC's injury in IgAN. Injury of TECs in IgAN is mainly associated with protein filtration from filtration barrier damage and mesangial–tubular crosstalk, and retinoic acid metabolism. The associated mechanism is shown in Figure 5.

Filtrated proteins including albumin, complement components, cytokines, growth factors, and Gd-IgA1 from endothelium injury and podocyte damage play an indisputable role in the tubulointerstitial injury. These filtrated proteins stimulate proximal tubular epithelial cells (PTECs) to secrete different kinds of inflammatory factors to build up an inflammatory micro-environment in the tubulointerstitium (5). Specifically, filtrated albumin could also stimulate TECs to secrete exosomes containing high levels of MCP-1 mRNA; then, the MCP-1 mRNA from TECs is directly transferred to macrophages in interstitium to enhance macrophage influx and induce tubulointerstitial inflammation (98). Plasmin is another kind of filtrated protein that could also induce damage to TECs, which is associated with strengthened macrophage and neutrophil infiltration (99). Injury of ETCs is usually accompanied by reduced reabsorption ability, in which the essential protein for proximal tubule reabsorption, megalin, is downregulated, under the effect of miR-148b (100).

Injury of TECs was also considered to be a result of mesangial–tubular crosstalk (5). The crosstalk between mesangial cells and TECs is mediated by TNF-α, TGF-β1, and MCP-1 (6). TNF-α from mesangial cells could enhance AT1R expression on TECs in the early phase and then boosts AT2R expression subsequently, after which increased Ang II level from mesangial cells could deteriorate TEC damage by inducing inflammatory response *via* AT1R through MAPK pathway and inducing TEC apoptosis *via* AT2R through caspase pathway (20). Furthermore, Ang II could also upregulate the expression of the mineralocorticoid receptor (MR) by PTECs, and binding of aldosterone to MR on PTECs induces cellular apoptosis through the generation of reactive oxygen species (101). TGF-β1 could increase the expression of NLRP3 on TECs transiently colocalizing with kidney injury molecule-1 (KIM-1) expression, which is correlated with the progression of IgAN (102). Lin et al. (103) found that KIM-1-positive TECs were associated with significantly increased infiltration of T cells and macrophages, probably through enhancing the expression of MCP-1 in TECs. PRR expressed on TECs plays a protective role in IgAN progression in which the expression level of PRR on TECs correlates with autophagy activation in TECs, suggesting that PRR might prevent TEC death and the subsequent fibrosis through activation of cytoprotective autophagic machinery (104).

Nakamura et al. (105) innovatively discovered that the “stimulated by retinoic acid 6” receptor (STRA6) and retinaldehyde dehydrogenase (RALDH) functioning in the uptake of retinol and converting retinol to retinoic acid (RA) are constitutively expressed on PTECs. The RA-producing capacity of PTECs is relocated to myofibroblasts in renal injury. The level of RA from myofibroblasts increases and RA promotes PTECs regeneration *via* RA receptor-γ in the nucleus. This investigation by Nakamura et al. (105) demonstrated the role of RA signaling in TEC repair and may suggest a beneficial effect of fibrosis in the early response to injury.

## 8. Conclusion

As the most common primary glomerulonephritis and the leading cause of kidney failure in the world, IgAN plays one of the dominant roles in affecting people's health and national socio-economic status. In this review, we debated the pathogenesis of IgAN by focusing on intrarenal inflammation initiated by Gd-IgA1-containing immune complex deposition with complement molecule activation affecting four main types of cells in nephrons, including mesangial cell, endothelial cell, podocyte, and tubular epithelial cells.

Activation of mesangial cells by deposition of Gd-IgA1-containing immune complexes with enhanced cellular proliferation, ECM expansion, and release of cytokines and chemokines plays a central role in deteriorating renal function in IgAN. Endothelium alteration in IgAN is mainly induced by Gd-IgA1 deposition and mesangial–endothelial crosstalk to strengthen the intrarenal inflammation. Podocyte damage in IgAN characterized by podocyte apoptosis and disorganization of SD is mainly induced by altered mesangial–podocyte crosstalk. Protein filtration into tubulointerstitium and mesangial–tubular crosstalk with inflammatory cell infiltration play the dominant role in TEC injury.

The mesangial cell might be another resource of Gd-IgA1 in addition to B cells. However, the role of Gd-IgA1 spontaneously produced by mesangial cells in enhancing the inflammatory response of mesangial cells and in deteriorating renal function in IgAN needs further laboratory investigation.

## Author contributions

All authors listed have made a substantial, direct, and intellectual contribution to the work and approved it for publication.
